# Parental psychosocial factors and children’s oral health-related quality of life: Data from a caries prevention study with phone-based support

**DOI:** 10.1186/s12903-025-05446-z

**Published:** 2025-01-18

**Authors:** Ida Brännemo, Anna Levinsson, Tove Hasselblad, Göran Dahllöf, Georgios Tsilingaridis

**Affiliations:** 1 Division of Pediatric Dentistry, Department of Dental Medicine, Karolinska Institutet, Stockholm, Sweden; 2 Center for Pediatric Oral Health Research, Stockholm, Sweden; 3 Department of Epidemiology, Biostatistics and Occupational Health, McGill University, Montreal, QC Canada; 4 Department of Social Medicine and Public Health, Sahlgrenska Academy, Gothenburg University, Gothenburg, Sweden; 5 Center for Oral Health Services and Research Mid-Norway, TkMidt, Trondheim, Norway

**Keywords:** Early childhood caries, General anesthesia, Health inequity, Parental support programs, Prevention

## Abstract

**Background:**

Caries burden in children disproportionately affects minority and socioeconomically disadvantaged populations. Severe early childhood caries requiring general anesthesia (GA) is a significant concern, with high caries relapse rates in subsequent years.

**Aim:**

To examine associations between parental psychosocial factors, children’s caries burden, and oral health-related quality of life (OHRQoL), including group differences, following a phone-based parental support intervention for children treated under GA for severe Early Childhood Caries (ECC).

**Methods:**

Data were collected during a randomized controlled trial examining a phone-based parental support program. Parental stress was assessed using the Swedish Parenthood Stress Questionnaire (SPSQ), dental fear was measured with the Corah Dental Anxiety Scale (CDAS), and dental attitudes were evaluated using the Dental Beliefs Survey (DBS-R). Children’s OHRQoL was assessed using the Parental-Caregivers Perception Questionnaire (P-CPQ) and the Family Impact Scale (FIS). The Total Observed Caries Experience index was used to calculate the child’s cumulative caries burden. Unadjusted analyses were performed using t-tests, with effect sizes calculated as Cohen’s d. A two-step path analysis examined how parental factors, intervention, and baseline caries influenced caries burden at a one-year follow-up, while also assessing caries burdens impact on children's well-being.

**Results:**

At the one-year follow-up, the intervention group demonstrated significantly improved OHRQoL with lower mean scores on the P-CPQ and FIS compared to the control group, indicating fewer oral health issues and less impact on family life. No statistical differences were found in parental stress, dental fears, or dental beliefs between groups. Path analysis identified baseline caries and treatment group as significant predictors of caries burden, while intervention group predicted better OHRQoL outcomes. The model explained 86.2% of caries burden variance and 12.9% of OHRQoL variance.

**Conclusion:**

A phone-based parental support program significantly improved children’s OHRQoL, even with a higher caries burden in the intervention group at the one-year follow-up. Parental stress, dental fears, and attitudes showed no differences and did not predict caries burden. Preventing early childhood caries in high-risk groups remains challenging, highlighting the importance of preventive initiatives that empower parents and foster collaboration with key stakeholders to reduce severe ECC.

Registered at https://clinicaltrials.gov/ Identifier: NCT02487043.

**Supplementary Information:**

The online version contains supplementary material available at 10.1186/s12903-025-05446-z.

## Introduction

The caries burden in children is skewed, disproportionally affecting children from minority populations and disadvantaged socioeconomical groups [1]. Previous studies have shown that caries prevention in high-risk populations is especially challenging [2–4]. Having severe caries disease at an early age may negatively affect a child’s quality of life in terms of pain causing difficulties eating and sleeping, resulting in reduced growth or limiting participation in everyday activities [5]. Young children treated under general anesthesia (GA) for severe early childhood caries (ECC) is a known high-risk group with relapse rates ranging between 20 and 80% in the succeeding years [6, 7]

Parental psychological factors, knowledge, and beliefs play an important role in shaping the choices and behaviors they model for their children, impacting oral health outcomes [8, 9]. Parental stress can affect a child’s oral health negatively by diminishing parents' ability to engage in consistent preventive practices [10]. Similarly, parental dental anxiety has been linked to dental avoidance and untreated decay in children [11]. A lack of perceived control over oral health issues, as seen in parents with an external locus of control, further reduces the likelihood of engaging in preventive care [8]. As parents bear the responsibility for their children’s oral health, it is important to consider these factors in caries prevention efforts, alongside biological causes.

Personal interventions, including telephone counseling, have been identified as possible measures for caries prevention by enhancing caregivers’ health literacy and self-efficacy, thus supporting their ability to make positive changes in their children's oral health [12]. In a previous publication, we describe a randomized controlled trial (RCT) focusing on children treated under GA for severe ECC [13]. In summary, it used a patient-family centered approach [14] built on the principles of Motivational Interviewing (MI) [15], and the trial intervention offered bi-weekly phone support by a dental nurse who was trained as an oral health coach to provide oral health education and support. Results from the study, namely the difficulties achieving the intended parental participation in the intervention as well as the high caries relapse rates in both the intervention and control groups, prompt further analysis of parental factors associated with caries progression over the study period. This study examines associations between parental psychometric factors, children’s caries burden, and oral health-related quality of life as well as treatment group differences following a phone-based parental support program.

## Methods

### Data collection

Data for this study were gathered during a randomized controlled trial (RCT) entitled “Phone-Based Parental Support Program for Caries Prevention in Children: A Randomized Controlled Trial” [13]. Detailed descriptions of the trial design and outcomes as well as study protocol have been published elsewhere, so only the salient features are described here. The study was approved by The Regional Ethics Review Board in Stockholm (Daybook no 2014/1863–31) and registered at https://clinicaltrials.gov/ Identifier: NCT02487043.

The primary aim of the RCT was to test a phone delivered, MI based parental support program aimed to reduce caries recurrence and improve oral health habits in children. Eligible participants were children under six years old, treated under GA for ECC, and with no other systemic diseases or developmental disorders and their parents. Following parental informed consent, 151 parent–child dyads were randomized to the intervention or the control group (usual care), allocation ratio 1:1, and 148 dyads participated in the study (74 in each randomization arm). The intervention group was offered planned biweekly phone counselling with an oral health coach for one year, accessible in Arabic, English, Polish, Turkish, and Swedish. Parents in the control group received standard advice on toothbrushing and sugar reduction. Both groups had equal access to dental care during the intervention period, managed by the child’s primary dental team.

### Study measures

Caries was measured using the six-grade diagnostic system International Caries Detection and Assessment System (ICDAS) [16] and examined by calibrated pediatric dentists blinded to group assignment [13]. Collection of caries data occurred at baseline (before GA) and one- and two-years post-treatment. Caries progression was defined as manifest caries on a previously non cavitated tooth or surface. Caries burden was defined as accumulated number of teeth affected by manifest caries over time, calculated using the Total Observed Caries Experience index (TOCE) [17]. Caregiver oral health behaviors on behalf of their children, including daily tooth brushing with fluoride toothpaste and dietary practices, were recorded at baseline and after one year. Results on caries progression and oral health habits have been previously published [13].

Parents completed psychometric questionnaires at their first meeting with the oral health coach and after one year. Parental stress was assessed by the Swedish Parenthood Stress Questionnaire (SPSQ) [18], dental fear was assessed by the Corah Dental Anxiety Scale (CDAS) [19], and dental attitudes and beliefs were assessed by The Dental Beliefs Survey (DBS-R) [20] and the Child’s OHRQoL [21, 22] using the Parental-Caregivers Perception Questionnaire (P-CPQ) and the Family Impact Scale (FIS). Table 1 lists the names of each scale and subscale, the number of questions included in each, a brief description of what each scale or subscale measures, and the score range for each. All scales have previously been used in a Swedish setting [18, 23–25]. For non-native Swedish speakers, language-adapted versions of the questionnaires were available, translated by professional interpreters and checked for errors by the bilingual health coaches. The oral health coaches assisted the completion when necessary.

**Table 1:** Description of parental psychometric questionnaires

Measuring instrument	Description	Response range
**Swedish Parenthood Stress Questionnaire (SPSQ)**^**a**^ Five dimensions: Incompetence (11 items) Role restriction (7 items) Social isolation (7 items) Spouse relationship problems (5items) Health problems (4 items)	Experienced parental stress and perceived strain	1–5 Parents mark the degree that they agree or disagree. Higher scores indicate greater stress
**Corah Dental Anxiety Scale (CDAS)**^**b**^ (4 items)	Level of anxiety that individuals may experience in relation to dental situations	1–5 1 (no anxiety) to 5 (extreme anxiety)
**The Dental Beliefs Survey (DBS-R)**^**c**^ Three dimensions: Ethics (11 items) Communication (9 items) Lack of control (8 items)	Patient’s perceptions about dentist’s behavior and the process of how dental care is delivered	1–5 Ranging from “never” (1) to “nearly always” (5). Higher scores indicate more negative beliefs about dentistry
**Oral Health Related Quality of Life (OHRQoL)**^d^ Two subscales: *The Parental-Caregivers Perception Questionnaire (P-CPQ, 33 items)* Oral symptoms (7 items) Functional limitations (7 items) Emotional well-being (9 items) Social well-being (10 items) *The Family Impact Scale (FIS, 14 items)* Parental and family activities (5 items) Parental emotions (4 items) Family conflict (4 items) Financial burden (1 item) Two global questions concerning oral health and general well-being	How oral health affects general functional and psychosocial well-being The Parental-Caregivers Perception Questionnaire (P-CPQ) provides opportunity to study OHRQoL in younger children who are unable to answer questions The Family Impact Scale (FIS) measures the effect of a child’s oral health on the family	0–4 “never” (0) to “every day or almost every day” (4) A “Don’t know” response option was also provided Global questions scored 1–5 #1 “excellent” (1) to “bad” (5) #2 “not at all” (1) to “very much” (5)

^a^Swedish Parenthood Stress Questionnaire (SPSQ): total score calculated as the mean of all items, scores for the five subscales as the mean of subscale items, ranging from 1 to 5, with higher scores indicating more stress [18]

^b^Corah Dental Anxiety Scale (CDAS): scores calculated as the sum of item responses, range 4–20, with a higher score indicating a higher dental fear and anxiety level [19]

^c^The Dental Beliefs Survey (DBS-R): scores calculated as the sum of item responses. The outcome is a sum of scores ranging from 28 (highly positive) to 140 (highly negative) [23]

^d^Oral Health-Related Quality of Life (OHRQoL): total score calculated as sum of all item scores, and subscale scores calculated as the sum of discrete subsets of items within the dimensions (P-CPQ range 0–132, FIS range 0–56). Higher scores indicate a greater negative impact on the child’s oral health-related quality of life, reflecting more problems such as pain, functional limitations, and emotional or social difficulties [27]

Sociodemographic characteristics were collected at baseline [13]. Families were classified according to the residential area-based caries risk measure used in the Stockholm Region (Health Need Area, HNA), where HNA 1–2 indicates a low caries risk and HNA 3–4 indicates a high risk [26].

### Statistical analyses

For the SPSQ, missing values were not imputed [28]. Total and subscale scores were calculated as the mean of answered items, conditional on less than 20% of the items in a subscale being missing. In the case of the CDAS, a single missing response was replaced with the mean of the completed CDAS items for that individual. For the DBS-R, missing items were substituted with the mean of the non-missing items on the subscale as long as no more than two items were missing [23]. For the OHRQoL, if fewer than 30% of the responses were “Don't know” or missing, these responses were replaced with the sample mean item score [21, 24]. Scales or subscales with missing responses exceeding the specified thresholds for each measure were excluded from the analysis.

Adherence to oral health beneficial dietary habits was calculated as the number of “no” responses to the following statements: “Meals/snacks > 5” (child has more than five meals or snacks per day), “Sweet drinks” (child consumes sweet drinks between meals), and “Sweets for snack” (child consumes sweet snacks at least three times per week).

Continuous and categorical variables, by treatment group at baseline and the one-year follow-up, were presented as mean and standard deviation or count and percentage, as appropriate.

Differences in measures between randomization arms and within randomization arms over time were calculated as Cohen’s d using the pooled standard deviation and the sample standard deviation of the mean difference, respectively. Statistical significance of the differences was evaluated using t-tests. The magnitude of change was represented by effect sizes, with values less than 0.2 indicating a small clinically meaningful change, 0.2 to 0.7 a moderate change, and greater than 0.8 a large change [29].

Internal consistency for scales and subscales was assessed using Cronbach’s alpha. Alpha coefficient between 0.70 and 0.80 was regarded as satisfactory and less than 0.5 as not acceptable [30].

A two-step path model was hypothesized to estimate the impact of parental factors and baseline child caries experience on children’s caries burden after the intervention (one-year follow-up). The analysis also evaluated the impact on children’s well-being, considering the potential influence of their one-year caries burden.

The first step examined SPSQ, DBS-R, CDAS, treatment group, and TOCE at baseline as predictors of children’s caries burden at the one-year follow-up. The second step examined the relationship between children’s caries burden at the one-year follow-up and their quality of life, measured by overall OHRQoL, P-CPQ sum score, and FIS sum score. CDAS at baseline was also included as a potential predictor, reflecting the possible influence of parental dental anxiety on perceptions of the child’s oral health-related quality of life [31]. Both steps of the path analysis were adjusted for parental background, health need area, and parental age, while toothbrushing habits and dietary habits were adjusted only for TOCE at the one-year follow-up.

Path analysis model fit was evaluated using confirmatory factor analysis, with a good model fit defined as; 1) chi-square (χ^2^) *p*-value > 0.05 (null hypothesis of a perfect fit cannot be rejected); 2) Root Mean Square Error of Approximation (RMSEA) < 0.05; 3) Comparative Fit Index (CFI) > 0.90; and 4) Standardized Root Mean Square Residual (SRMR) < 0.08. [32]. All statistical analyses were carried out using SPSS version 28 and R version 4.2.1.

## Results

### Final population and background characteristics

Of the 148 caregiver-child dyads in the RCT, 109 dyads (73.6%) had complete baseline and Year 1 data for path analysis. Table 2 presents descriptive statistics for the sample’s caries burden and background variables. More than half of the children were boys, and most of the adult respondents were female. All children presented with a high total caries burden at baseline. The caries burden in the intervention group after one year was significantly higher than that in the control group. No statistically significant differences were detected between individuals included in the path model and those excluded due to incomplete data for at least one variable (Appendix Table 1).

**Table 2:** Descriptive statistics of caries burden and socio-demographic variables for the sample (*n* = 109)

	**Intervention group** ***n* = 52**	**Control group** ***n* = 57**	***P*-value** group difference
**Variable**	**Mean (SD) / N (%)**	**Mean (SD) / N (%)**	
Age – respondent (years)	35.37 (6.65)	34.67 (6.80)	0.590
Age – child (months)	54.96 (11.73)	52.74 (12.22)	0.335
Sex – respondent			0.457
Woman	39 (75.0%)	38 (66.7%)	
Man	13 (25.0%)	19 (33.3%)	
Sex – child			0.172
Girl	17 (32.7%)	27 (47.4%)	
Boy	35 (67.3%)	30 (52.6%)	
HNA			0.555
1–2	29 (55.8%)	36 (63.2%)	
3–4	23 (44.2%)	21 (36.8%)	
Parental background^a^			0.234
Sweden	7 (13.5%)	13 (22.8%)	
Europe	14 (26.9%)	9 (15.8%)	
Rest of world	31 (59.6%)	35 (61.4%)	
TOCE baseline	12.37 (3.54)	11.40 (3.75)	0.173
TOCE 1-year	14.23 (3.25)	12.77 (3.67)	0.031*
Oral health beneficial dietary habits			0.860
0	17 (32.7%)	18 (31.6%)	
1	13 (25.0%)	19 (33.3%)	
2	17 (32.7%)	13 (22.8%)	
3	5 (9.6%)	7 (12.3%)	
Daily toothbrushing frequency			0.981
0x	22 (42.3%)	25 (43.9%)	
1x	9 (17.3%)	10 (17.5%)	
2x	21 (40.4%)	22 (38.6%)	

All sociodemographic variables were collected at baseline

*HNA* health need area – residential area mapped as high-risk caries areas (HNA 3–4) or low caries risk areas (HNA 1–2), *TOCE* Total Observed Caries Experience

^a^Based on reported native language

^*^: *p*-value < .05

Missing data for each instrument are reported in Table 3 with detailed information provided in Appendix Additional file 1.

**Table 3:** Descriptive statistics for continuous scale scores by treatment group

			**Intervention group**		**Control group**		***p*-value†**	**Cronbach’s alpha**
**Scale**	**Timepoint**	**Score**	Mean (SD)	Missing (%)	Mean (SD)	Missing (%)		
**SPSQ**	Baseline	Overall	2.30 (0.53)	0 (0.0%)	2.35 (0.58)	0 (0.0%)	0.575	0.84
	Baseline	Incompetence	2.09 (0.71)	0 (0.0%)	2.21 (0.73)	0 (0.0%)	0.301	0.75
	Baseline	Role restriction	3.18 (0.98)	0 (0.0%)	3.31 (1.03)	0 (0.0%)	0.466	0.80
	Baseline	Social isolation	1.92 (0.72)	0 (0.0%)	1.74 (0.67)	0 (0.0%)	0.126	0.60
	Baseline	Spouse relationship problems	2.20 (0.80)	1 (1.4%)	2.30 (0.80)	0 (0.0%)	0.406	0.40
	Baseline	Health problems	2.20 (0.85)	0 (0.0%)	2.25 (0.85)	0 (0.0%)	0.717	0.60
	1-year f-u	Overall	2.02 (0.49)	15 (20.3%)	2.19 (0.56)	9 (12.2%)	0.075	0.87
	1-year f-u	Incompetence	1.81 (0.56)	15 (20.3%)	1.99 (0.64)	9 (12.2%)	0.114	0.75
	1-year f-u	Role restriction	2.76 (1.02)	15 (20.3%)	3.07 (0.94)	9 (12.2%)	0.081	0.81
	1-year f-u	Social isolation	1.69 (0.58)	15 (20.3%)	1.74 (0.66)	9 (12.2%)	0.675	0.58
	1-year f-u	Spouse relationship problems	2.00 (0.70)	15 (20.3%)	2.10 (0.80)	9 (12.2%)	0.324	0.51
	1-year f-u	Health problems	1.91 (0.75)	15 (20.3%)	2.12 (0.84)	9 (12.2%)	0.151	0.68
**CDAS**	Baseline	Sum score	9.00 (4.00)	0 (0.0%)	10.00 (4.00)	0 (0.0%)	0.052	0.91
	1-year f-u	Sum score	7.21 (2.87)	15 (20.3%)	8.31 (3.35)	9 (12.2%)	0.055	0.90
**DBS-R**	Baseline	Overall	50.28 (21.99)	0 (0.0%)	52.77 (20.34)	0 (0.0%)	0.475	0.93
	Baseline	Ethics	21.90 (9.60)	0 (0.0%)	23.20 (9.60)	0 (0.0%)	0.406	0.83
	Baseline	Communication	14.77 (7.45)	0 (0.0%)	15.38 (6.89)	0 (0.0%)	0.607	0.86
	Baseline	Lack of control	14.00 (7.00)	0 (0.0%)	14.00 (7.00)	0 (0.0%)	0.615	0.86
	1-year f-u	Overall	42.46 (15.75)	15 (20.3%)	46.04 (19.22)	9 (12.2%)	0.261	0.93
	1-year f-u	Ethics	18.05 (7.77)	15 (20.3%)	18.82 (8.44)	9 (12.2%)	0.596	0.85
	1-year f-u	Communication	13.00 (5.00)	15 (20.3%)	15.00 (8.00)	9 (12.2%)	0.075	0.88
	1-year f-u	Lack of control	11.61 (5.07)	15 (20.3%)	12.37 (6.10)	9 (12.2%)	0.454	0.88
**OHRQoL**	Baseline	Overall OHRQoL	39.42 (25.12)	1 (1.4%)	41.44 (20.71)	2 (2.7%)	0.599	0.93
	Baseline	Global score 1	4.16 (1.07)	0 (0.0%)	4.18 (0.98)	0 (0.0%)	0.926	N/A
	Baseline	Global score 2	2.86 (1.31)	0 (0.0%)	2.98 (1.19)	0 (0.0%)	0.554	N/A
	1-year f-u	Overall OHRQoL	17.56 (12.74)	18 (24.3%)	28.89 (18.85)	10 (13.5%)	<.001*	0.92
	1-year f-u	Global score 1	3.00 (1.00)	16 (21.6%)	3.00 (1.00)	9 (12.2%)	0.025*	N/A
	1-year f-u	Global score 2	2.00 (1.00)	16 (21.6%)	3.00 (1.00)	9 (12.2%)	0.400	N/A
PCPQ	Baseline	Overall	22.21 (17.69)	1 (1.4%)	25.36 (16.36)	2 (2.7%)	0.267	0.92
	Baseline	Oral symptoms	9.05 (5.77)	0 (0.0%)	10.02 (6.00)	1 (1.4%)	0.316	0.78
	Baseline	Functional symptoms	5.44 (5.18)	1 (1.4%)	7.08 (5.32)	1 (1.4%)	0.061	0.74
	Baseline	Emotional symptoms	4.78 (5.89)	1 (1.4%)	5.53 (5.34)	2 (2.7%)	0.424	0.85
	Baseline	Social well-being	2.91 (4.42)	1 (1.4%)	2.77 (3.27)	2 (2.7%)	0.839	0.84
	1-year f-u	Overall	9.41 (9.08)	18 (24.3%)	17.15 (13.86)	10 (13.5%)	<.001*	0.90
	1-year f-u	Oral symptoms	3.70 (3.35)	17 (23.0%)	6.23 (4.74)	9 (12.2%)	<.001*	0.76
	1-year f-u	Functional symptoms	3.41 (3.83)	18 (24.3%)	5.92 (5.54)	10 (13.5%)	0.004*	0.78
	1-year f-u	Emotional symptoms	1.05 (2.06)	18 (24.3%)	3.23 (4.34)	10 (13.5%)	<.001*	0.79
	1-year f-u	Social well-being	1.18 (2.12)	18 (24.3%)	1.82 (2.43)	10 (13.5%)	0.128	0.73
FIS	Baseline	Overall	10.18 (8.47)	1 (1.4%)	8.95 (5.41)	2 (2.7%)	0.297	0.79
	Baseline	Parental and family activities	4.55 (4.10)	0 (0.0%)	4.46 (2.55)	2 (2.7%)	0.880	0.61
	Baseline	Parental emotions	4.27 (3.68)	0 (0.0%)	3.19 (2.58)	2 (2.7%)	0.042*	0.66
	Baseline	Family conflict	1.25 (2.47)	1 (1.4%)	1.11 (2.05)	2 (2.7%)	0.719	0.74
	Baseline	Financial burden	0.14 (0.60)	0 (0.0%)	0.18 (0.53)	0 (0.0%)	0.633	N/A
	1-year f-u	Overall	2.83 (3.50)	18 (24.3%)	5.83 (6.47)	10 (13.5%)	0.002*	0.82
	1-year f-u	Parental and family activities	1.58 (1.99)	16 (21.6%)	2.81 (3.36)	9 (12.2%)	0.014*	0.67
	1-year f-u	Parental emotions	1.17 (1.80)	16 (21.6%)	2.11 (2.70)	9 (12.2%)	0.024*	0.71
	1-year f-u	Family conflict	0.32 (0.82)	18 (24.3%)	0.82 (1.68)	10 (13.5%)	0.037*	0.54
	1-year f-u	Financial burden	0.00 (0.00)	16 (21.6%)	0.00 (0.00)	9 (12.2%)	0.902	N/A

*CDAS* Corah Dental Anxiety Scale, *DBS-R* Dental Beliefs Survey, *FIS* Family Impact Scale, *f-u* follow-up, *OHRQoL* Oral Health-Related Quality of Life, *P-CPQ* Parental-Caregiver Perceptions Questionnaire, *SD* standard deviation, *SPSQ* Swedish Parenthood Stress Questionnaire

^*^p-value < . 05; † *p*-value from t-test of equality of means between groups

## Outcomes

### Comparison of treatment groups and changes over time

Table 3 presents the mean scores for each instrument at baseline and the one-year follow-up by treatment group. A comparison of scale and subscale scores between the treatment groups revealed no statistical differences in parental stress (SPSQ), dental fear (CDAS), or dental beliefs (DBS-R). However, we found several statistically significant differences in the child’s oral health-related quality of life (OHRQoL). At the one-year follow-up, the intervention group reported significantly lower mean scores for the overall P-CPQ and three of its subscales (Oral Symptoms, Functional Symptoms, and Emotional Symptoms) as well as for the overall FIS and three of its subscales (Parental and Family Activities, Parental Emotions, and Family Conflict) compared to the control group. These lower scores reflect a reduced negative impact on the child’s OHRQoL, indicating fewer oral health-related problems and a lessened impact on family life. No significant differences in scale scores were found between children who developed additional caries, defined as caries on a previously non-cavitated surface, and those without caries relapse at the one-year follow-up (data not shown).

Cronbach’s alpha, calculated for each scale and subscale, showed that internal consistency was satisfactory (> 0.70) for all overall scale scores and lower but still acceptable for many subscales, except for SPSQ for the subscale social isolation (α = 0.40), at baseline (Table 3).

**Fig. 1:**
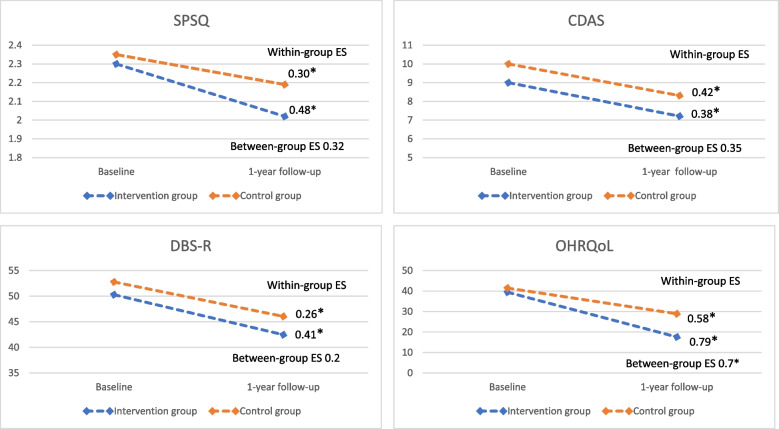
Scale score Improvements over time. The x-axis represents time points pre and post intervention and the y-axis represents the group mean score. Note: * *p* < 0.05. CDAS: Corah Dental Anxiety Scale: DBS-R: Dental Beliefs Survey; ES: Effects size; OHRQoL: Oral Health Related-Quality of Life (includes Global scores, Family Impact Scale and The Parental-Caregivers Perception Questionnaire); SPSQ: Swedish Parenthood Stress Questionnaire

For both groups, all total scale scores were lower post-intervention than pre-intervention (Fig. 1). Low to moderate effect sizes were detected for both between- and within-group differences, with OHRQoL showing the greatest improvement (Cohen’s d = 0.7 for between-group difference, d = 0.79 within the intervention group, and d = 0.58 within the control group). Within the intervention group, statistically significant improvement over time was detected for all but three subscales (SPSQ Social isolation, OHRQoL Global score 2, and FIS Financial burden). The control group showed less consistent improvement although statistically significant differences between timepoints were detected for all total scale scores (Appendix Table 2).

As sensitivity analyses of the intervention effect on improvement of overall scores between baseline and 1-year, we investigated the association between treatment intensity (i.e. time spent on the phone with the OHC) and change in scores using linear regression, controlling for baseline scores. The association was not statistically significant for any scores (Appendix Table 3.).

### Path analysis of predictors for caries burden and oral health-related quality of life

**Fig. 2:**
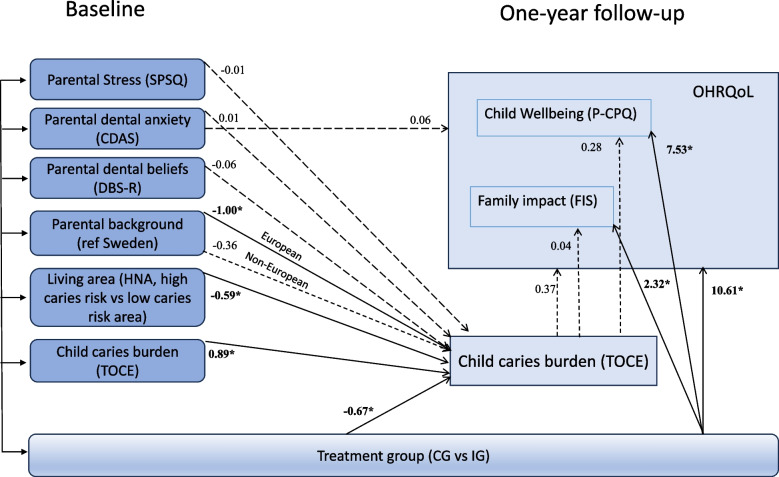
A path analysis was conducted in two steps to estimate the effects of parental factors, treatment group, and baseline child caries experience on the children’s caries burden after the intervention (one-year follow-up). The analysis also assessed the impact on the children’s well-being, considering the potential influence of their one-year caries burden. β values indicate the direction and magnitude of the relationship. Links with solid lines represent a significance level of *p*** < **0.05*. Both steps of the path analysis were adjusted for parental background, health need area, and parental age, while toothbrushing habits and dietary habits were adjusted only for TOCE at the one-year follow-up. However, for clarity, parental background and health need area are only depicted in the figure for the first step, as they emerged as statistically significant predictors. CDAS: Corah Dental Anxiety Scale; CG: control (standard treatment) group; DBS-R: Dental Beliefs Survey; FIS: Family Impact Scale; HNA: health need area; IG: intervention (oral health coach) group; OHRQoL: Oral Health-Related Quality of Life; P-CPQ: Parental-Caregiver Perceptions Questionnaire; SPSQ: Swedish Parenthood Stress Questionnaire; TOCE: total observed caries experience

In the first step of the path model, none of the psychometric scales – i.e., parental stress, dental fears, or dental attitudes and beliefs – were statistically significant predictors. However, baseline caries (β = 0.89, 95% confidence interval [CI] 0.81–0.96) and treatment group (control vs intervention, β = ** − **0.67, 95% CI − 1.19 to − 0.15) emerged as significant predictors of caries burden at the one-year follow-up. Significant adjustment variables also included parental background and living area (HNA) with a negative association between caries burden at the one-year follow-up and respondents with European background compared to those with Swedish background as well as a negative association between caries burden at the one-year follow-up and high-risk HNA compared to low-risk HNA (Table 4). Parental background and living area (HNA) were incorporated into the first step of the final model (Fig. 2).

**Table 4:** Path model analyses of overall OHRQoL, P-CPQ, and FIS at the one-year follow-up, *N* = 109

Model	Outcome	Predictor	Estimate	95% CI	*p*-value
a, b, c	TOCE 1-year	SPSQ baseline	-0.01	-0.52–0.50	0.963
		DBS-R baseline	0.01	-0.01–0.02	0.394
		CDAS baseline	-0.06	-0.12–-.01	0.076
		TOCE baseline	0.89	0.81–0.96	< .001*
		Treatment group (CG vs IG)	-0.67	-1.19–-.15	0.012*
		Parental background (ref = Swe native)			
		*Europe*	-1.00	-1.86–-.14	0.022*
		*Rest of world*	-0.36	-1.13–0.41	0.360
		HNA (3,4 vs 1,2)	-0.59	-1.14–-.05	0.033*
		Age respondent	-0.01	-0.04–0.03	0.763
		Dietary behavior	-0.26	-0.53–0.01	0.058
		Daily toothbrushing frequency (ref = 0)			
		*Once daily*	0.48	-0.24–1.20	0.189
		*Twice daily*	0.36	-0.26–0.98	0.259
a	OHRQoL 1-year	CDAS baseline	0.06	-0.68–0.80	0.871
		TOCE 1-year	0.37	-0.54–1.27	0.429
		Treatment group (CG vs IG)	10.61	4.35–16.87	0.001*
		Parental background (ref = Swe native)			
		*Europe*	-3.03	-12.95–6.90	0.550
		* Rest of world*	1.63	-6.96–10.22	0.710
		HNA (3,4 vs 1,2)	-1.92	-8.31–4.46	0.555
		Age respondent	-0.08	-0.53–0.38	0.732
b	P-CPQ 1-year	CDAS baseline	0.94	-0.45–0.63	0.413
		TOCE 1-year	0.28	-0.39–0.94	0.732
		Treatment group (CG vs IG)	7.53	2.95–12.11	0.001*
		Parental background (ref = Swe native)			
		*Europe*	-1.12	-8.38–6.15	0.763
		*Rest of world*	1.27	-5.01–7.56	0.691
		HNA (3,4 vs 1,2)	-0.65	-5.32–4.02	0.784
		Age respondent	-0.05	-0.38 – 0.29	0.785
c	FIS 1-year	CDAS baseline	0.01	-0.26–0.23	0.910
		TOCE 1-year	0.04	-0.26–0.34	0.800
		Treatment group (CG vs IG)	2.32	0.25–4.38	0.028*
		Parental background (ref = Swe native)			
		*Europe*	-1.80	-5.08–1.47	0.281
		*Rest of world*	-0.35	-3.18–2.48	0.809
		HNA (3,4 vs 1,2)	-1.96	-4.07–0.14	0.068
		Age respondent	-0.04	-0.19–0.11	0.594

Model fit a: χ^2^ p-value = 0.886, RMSEA =  < .001, CFI = 1.000, SRMR = 0.013

Model fit b: χ^2^ p-value = 0.936, RMSEA =  < .001, CFI = 1.000, SRMR = 0.012

Model fit c: χ^2^ p-value = 0.521, RMSEA =  < .001, CFI = 1.000, SRMR = 0.019

Variables age respondent, HNA, Parental background, Dietary behavior, and Daily toothbrushing frequency were measured at baseline

*CDAS* Corah Dental Anxiety Scale, *CI* confidence interval, *CG* control (standard treatment) group, *DBS-R* Dental Beliefs Survey, *FIS* Family Impact Scale, *HNA* health need area, *IG* intervention (oral health coach) group, *OHRQoL* Oral Health-Related Quality of Life, *P-CPQ* Parental-Caregiver Perceptions Questionnaire, *SPSQ* Swedish Parenthood Stress Questionnaire, *TOCE* total observed caries experience

^*^p-value < .05

In the second step (i.e., analyzing predictors of overall OHRQoL, P-CPQ, and FIS at the one-year follow-up), treatment group was consistently identified as the only statistically significant predictor, favoring the intervention group (control vs intervention, OHRQoL β = 10.61, 95% CI 4.35–16.87; P-CPQ β = 7.53, 95%CI 2.95–12.11; FIS β = 2.32, 95% CI 0.25–4.38) (Fig. 2, Table 4).

Variance explained by the model (unadjusted R^2^) was 86.2% for TOCE, indicating a strong predictive capacity for caries burden. In contrast, the model explained only 12.9% of the variance in overall OHRQoL, 10.5% in overall P-CPQ, and 11.5% in overall FIS at the one-year follow-up, suggesting that it accounts for a relatively small portion of the variability in children's oral health-related quality of life and family impact.

All three path models – using overall OHRQoL, overall P-CPQ, and overall FIS scores, respectively, at one-year follow-up as final outcome – fulfilled model fit criteria. The lowest SRMR was detected for the P-CPQ path (SRMR = 0.012), however the difference to the overall OHRQoL path (SRMR = 0.013) was minute.

Missing values in responses to psychometric measures questionnaires were partly imputed as described in the methods section. However, comparison between path model analysis result using imputed and only complete data showed that imputation maintained the integrity of the results.

## Discussion

### Main findings

This study explored possible associations between children’s caries burden, oral health-related quality of life, and parental psychosocial factors as well as group differences following a phone-based parental support intervention. Significant improvements in oral health-related quality of life were observed for the intervention group receiving a phone-based parental support program at the one-year follow-up compared to the control group. These improvements included fewer oral symptoms, reduced functional limitations, and a less negative impact on family life. Despite the intervention group having a higher caries burden at the one-year follow-up, being in the intervention group predicted a significant positive contribution to oral health-related quality of life. Parental stress, dental fears, and attitudes did not differ between groups and were not significant predictors of caries burden after one year.

## Findings in relation to previous research

### Parental stress

Parental stress can influence children’s health by both increasing children’s stress and affecting parenting behaviors [33]. Parents preoccupied with more immediate concerns may find it difficult to maintain preventive oral health practices for their children [10, 34]. Only a limited number of studies, employing various tools, have examined the relationship between parental stress and ECC [35]. In our study, we employed the Swedish Parenthood Stress Questionnaire (SPSQ), a revised version of the American Parenting Stress Index (PSI) [36]. Our results align with findings that observed no significant correlation between total parental stress using PSI and ECC, although specific domains, such as child reinforcement and life stress, showed variations [37]. Conversely, other studies have identified parental stress as a significant predictor of ECC, particularly using the PSI-Short Form [34, 38, 39] Interestingly, one study reported an inverse association, suggesting that higher maternal stress could be protective against caries [40]. To our knowledge, the SPSQ has not been used to assess parental stress in relation to severe ECC, but Swedish data for parents of children with rare disorders aged 0–6 years [41] show comparable stress levels to our sample, except for higher scores in the social isolation domain.

### Dental fear and anxiety

Our study did not reveal a significant impact of parental dental anxiety, as measured by the Corah Dental Anxiety Scale (CDAS), on child OHRQoL or caries burden, even though both groups showed a significant within-group improvement over time. This finding contrasts with previous studies that have demonstrated a correlation between parental dental anxiety and poorer OHRQoL [31, 42] as well as untreated caries in preschool children [11]. Both groups experienced increased caries burden over time, with the higher CDAS values at baseline likely influenced by concerns regarding the child’s dental treatment under GA.

### Dental beliefs

A life course model of oral health found that childhood socioeconomic status and parental oral health-related beliefs were linked to participants’ dental beliefs in adolescence and early adulthood, highlighting the role of parents in fostering positive oral health habits over time and their influence on developing effective dental self-care behaviors [9]. The DBS-R, used in this study, has been validated as a reliable instrument for assessing attitudes toward dentists and dental care across various Swedish clinical and nonclinical populations. With a total score > 42 indicating more negative attitudes toward dental care [23]. In our study, baseline values were elevated in both groups, indicating negative perceptions of dental care. However, parental attitudes did not serve as predictors for the child’s caries development. Over time, the scores normalized toward a mean of 42, showing significant improvements in both groups, with a greater within-group improvement in the intervention group.

### Oral health-related quality of life

Previous research has demonstrated that dental treatment of ECC performed under general anesthesia has an immediate positive effect on children's well-being and family dynamics [24]. This finding aligns with our study, which observed improvements for both groups over time, likely due primarily to the relief of pain and discomfort resulting from the dental treatment itself. Thus, the severity of the dental condition leading to treatment under general anesthesia may influence the outcomes, with the potential for improvement varying according to the degree of severity. Interestingly, despite the intervention group having a higher caries burden at the one-year follow-up, they reported significant improvements in the child’s OHRQoL, with an effect size of 0.7 for between-group comparisons. This enhancement in the intervention group could be attributed to the support received from the oral health coaches, which may have fostered a sense of confidence among parents. Our earlier reports indicate that this group experienced increased confidence during the intervention period [13]. Moreover, it is plausible that the intervention helped parents perceive dental-related challenges as less demanding. By creating a supportive environment, parents may have managed their children’s dental issues more positively, potentially alleviating the impact within the family. This shift in perspective may explain why parents in the intervention group reported better OHRQoL despite their children’s higher caries burden. A previous study involving children at high risk for caries found that higher parental self-efficacy was linked to improvements in oral health knowledge but did not correlate with reduced caries progression or improved oral health behaviors [43]. Silva et al. also emphasized the role of parental well-being and emotional regulation in significantly influencing the reported well-being of children [44].

## Methodological considerations

This study presents several potential limitations. Psychosocial factors were assessed through parent-reported scores, which reflect hypothetical constructs such as parenting stress, dental fear, and attitudes, making them indirect measures of exposures among children. Due to this indirect nature, associations with such scores should be interpreted cautiously, particularly regarding the mechanisms involved. Additionally, in terms of OHRQoL, parents may not always be fully aware of their children’s activities and feelings; however, for the age groups studied, parental proxy ratings are necessary. Given that parents are responsible for their children's care, their perceptions play a crucial role in decisions regarding oral health and healthcare utilization [21]. Furthermore, comparisons of scale scores between studies must be approached carefully, as findings may be influenced by differences in national oral health contexts, geographic and cultural factors, sample selection, and the timing of measurements.

Another limitation is the use of language-adapted questionnaires as the instruments were not fully validated through cross-cultural adaptation for all languages used in the study. To avoid excluding a significant portion of the target group, we chose to offer the instrument in languages other than Swedish, assisted by bilingual oral health coaches, reflecting the real-world situation where parents of children with severe ECC might be non-Swedish speaking. Estimates of the reliability of the overall scores indicated satisfactory internal consistency, with Cronbach’s alpha coefficients exceeding 0.70. However, the translation process may have affected the preservation of the original instrument’s validity across different languages and cultural contexts. We selected questionnaires previously used in similar studies and Swedish patient groups; however, the number of instruments and items was substantial. Other instruments such as the ECOHIS [45] for measuring OHRQoL have fewer items, which reduces participant burden and data collection costs.

Regarding sample size, the power calculation for the RCT focused on caries progression rather than the effect size of the psychometric instruments. Consequently, the sample size may have limited the ability to detect significant differences between groups, despite the intervention group demonstrating more consistent improvements. Furthermore, the group of children included in the path analysis was even more decimated, which may have further impacted the robustness of the findings.

In the path analysis, we chose to explore whether baseline parental factors, along with the child’s initial caries experience, could predict caries burden at the one-year follow-up. This approach was deemed most clinically meaningful to potentially identify children at high risk for future caries development. Consistent with our findings, baseline caries is a strong predictor of future caries development [46]. Preventing ECC in high-risk groups has proven especially challenging [2]. Notably, young children treated under general anesthesia for severe ECC represent a high-risk group, exhibiting high relapse rates, as observed in our previously published RCT [13]. A recent Swedish study explored health-promoting factors among caries-free young adults from low socioeconomic backgrounds, highlighting the important role parents play in providing guidance, support, and stability [47]. This emphasizes the need for initiatives that empower parents and promote collaboration with institutions such as preschools and child health services to prevent the establishment of severe ECC from the onset.

## Conclusion and future implications

In conclusion, this study demonstrates that a phone-based parental support program significantly enhanced children’s OHRQoL, despite the intervention group experiencing a higher caries burden at the one-year follow-up. Although parental stress, dental fears, and attitudes did not differ between groups and were not significant predictors of caries burden after one year, it is essential to recognize the critical role parents play in their children's oral health. Initiatives that empower parents and promote collaboration are essential to prevent ECC from the very beginning. Future research should focus on strengthening these collaborative efforts to support parents in managing their children's oral health proactively.

## Supplementary Information

Supplementary Material 1: Appendix Table 1 Descriptive statistics for *N*= 109 included in path model analyses and available data for *N*≤ 39 with incomplete data for path model variables. Appendix Additional file 1 Questionnaires missing data and imputation. Appendix Table 2 Effect sizes for between- and within-treatment group differences . Appendix Table 3 Sensitivity analyses: association between treatment intensity and change in questionnaire scores between baseline and 1-year follow-up, within the intervention group

## Data Availability

The datasets used and/or analyzed during the present study are available from the corresponding author upon reasonable request.

## Abbreviations

CDAS: Corah Dental Anxiety Scale
CFI: Comparative Fit Index
CG: Control Group
DBS-R: The Dental Beliefs Survey
ECC: Early Childhood caries
FIS: The Family Impact Scale
GA: General anesthesia
HNA: Health Need Area
ICDAS: International Caries Detection and Assessment System
IG: Intervention Group
MI: Motivational Interviewing
OHRQoL: Oral Health-Related Quality Of Life
P-CPQ: Parental-Caregivers Perception Questionnaire
RCT: Randomized controlled trial
RMSEA: Root Mean Square Error of Approximation
SPSQ: Swedish Parenthood Stress Questionnaire
SRMR: Standardized Root Mean Square Residual
TOCE: Total Observed Caries Experience
