# Supplementary material for: Parental psychosocial factors and children’s oral health-related quality of life: Data from a caries prevention study with phone-based support
Source: BMC Oral Health. 2025 Jan 18;25:94. doi: 10.1186/s12903-025-05446-z (PMC11742752; doi:10.1186/s12903-025-05446-z)
Supplement: Supplementary file 1 — Supplementary Material 1: Appendix Table 1 Descriptive statistics for N= 109 included in path model analyses and available data for N≤ 39 with incomplete data for path model variables. Appendix Additional file 1 Questionnaires missing data and imputation. Appendix Table 2 Effect sizes for between- and within-treatment group differences . Appendix Table 3 Sensitivity analyses: association between treatment intensity and change in questionnaire scores between baseline and 1-year follow-up, within the intervention group [file 12903_2025_5446_MOESM1_ESM.pdf]

APPENDIX

BMC Oral Health

Parental psychosocial factors and children's oral health-related quality of life: Data from a caries prevention study with phone-based support

Ida Brännemo[1,2], Anna Levinsson [3,4], Tove Hasselblad[1], Göran Dahllöf[1,2,5] and Georgios Tsilingaridis[1,2]

1. Division of Pediatric Dentistry, Department of Dental Medicine, Karolinska Institutet, Stockholm, Sweden

2. Center for Pediatric Oral Health Research, Stockholm, Sweden

3. Department of Epidemiology, Biostatistics, and Occupational Health, McGill University, Montreal, QC, Canada

4. Department of Social Medicine and Public Health, Sahlgrenska Academy, Gothenburg University, Gothenburg, Sweden

5. Center for Oral Health Services and Research Mid-Norway, TkMidt, Trondheim, Norway

Correspondence
Ida Brännemo
Division of Pediatric Dentistry
Department of Dental Medicine
Karolinska Institutet
141 86 Huddinge, Sweden

Phone: +46-8-524 880 39
E-mail: ida.brannemo@ki.se

APPENDIX

Appendix Table 1. Descriptive statistics for N= 109 included in path model analyses and available data for N≤ 39 with incomplete data for path model variables

		Excluded			Included		
**Variable (timepoint)**		Valid N	Mean	SD	Valid N	Mean	SD
SPSQ overall (bl)		39	2.45	0.59	109	2.29	0.54
CDAS (bl)		39	9.85	4.55	109	9.62	4.09
DBS-R (bl)		39	57.39	25.60	109	49.43	19.01
Overall OHRQoL (1y)		11	27.83	21.72	109	23.17	16.72
P-CPQ overall (1y)		11	16.42	15.54	109	13.25	12.14
FIS overall (1y)		11	5.23	6.16	109	4.35	5.44
TOCE (bl)		37	12.22	3.34	109	11.86	3.67
TOCE (1y)		13	13.15	3.67	109	13.47	3.54
Age respondent (bl)		31	36.00	8.03	109	35.00	6.71
Number of oral health-adverse dietary habits (bl)		39	0.92	1.04	109	1.17	1.01
HNA (bl)	1-2	22			65		
	3-4	17			44		
Parental background (bl)	Sweden	5			20		
	Europe	10			23		
	Rest of world	24			66		
Daily toothbrushing (bl)	0	16			47		
	1	13			19		
	2	10			43		

Note. *: p-value < .05

1y: one-year follow-up; bl: baseline; SD: standard deviation

APPENDIX

Additional file 1 Questionnaires missing data and imputation

For SPSQ, one individual in the intervention group was missing the score for subscale “spouse relationship problems” at baseline due to four out of five items missing. A total of 24 individuals (15 in the intervention group and 9 in the control group) did not answer the SPSQ questionnaire at the one-year follow-up, but no additional subscale scores were missing due to less than 80% of items answered.

For CDAS There were no individuals with missing data at baseline. At 1-year follow-up one intervention-group individual was missing the response for the first item, which was replaced by the mean of the three non-missing items for the calculation of the score. Fifteen individuals in the intervention group and nine individuals in the control group did not complete the questionnaire at the one-year follow up.

For DBS-R, four individuals had one response missing at baseline, which was replaced by the mean of the non-missing items on the subscale. At the one-year follow-up, 15 individuals in the intervention group and 9 individuals in the control group did not complete the questionnaire. Four individuals had one missing response, which was replaced by the mean of the non-missing items on the subscale.

For OHRQoL at baseline, one participant from the intervention group and two from the control group were excluded for having ≥ 30% “don’t know” or missing responses. At the one-year follow-up, 25 individuals (16 in the intervention group and 9 in the control group) did not complete the OHRQoL questionnaire, and three participants (two in the intervention group and one in the control group) were excluded for providing responses to < 70% of the items. Twenty-five participants in the intervention group and 29 in the control group were missing less than 15 item responses, and missing values were replaced with the sample mean item responses.

APPENDIX

Appendix Table 2 Effect sizes for between- and within-treatment group differences

Scale	Subscale / Group	Between-group effect sizes ⸸		Within-group effect sizes □
		Baseline	1-year follow-up	Baseline to 1-year follow-up
		ES (95% CI)	ES (95% CI)	ES (95% CI)
**SPSQ**	Overall	0.09 (-0.23–0.42)	0.32 (-0.03–0.68)	
	IG	N=74	N=59	0.48 (0.21–0.75)*
	CG	N=74	N=65	0.30 (0.05–0.55)*
	Incompetence	0.17 (-0.15–0.49)	0.29 (-0.07–0.64)	
	IG	N=74	N=59	0.33 (0.06–0.59)*
	CG	N=74	N=65	0.31 (0.06–0.56)*
	Role restriction	0.12 (-0.20–0.44)	0.32 (-0.04–0.67)	
	IG	N=74	N=59	0.43 (0.16–0.69)*
	CG	N=74	N=65	0.24 (-0.01–0.48)
	Social isolation	-0.25 (-0.58–0.07)	0.08 (-0.28–0.43)	
	IG	N=74	N=59	0.24 (-0.02–0.50)
	CG	N=74	N=65	0.06 (-0.19–0.30)
	Spouse relationship problems	0.14 (-0.19–0.46)	0.18 (-0.18–0.53)	
	IG	N=73	N=59	0.28 (0.02–0.54)*
	CG	N=74	N=65	0.12 (-0.13– 0.36)
	Health problems	0.06 (-0.26–0.38)	0.26 (-0.10–0.61)	
	IG	N=74	N=59	0.27 (0.01–0.53)*
	CG	N=74	N=65	0.13 (-0.11–0.38)
**CDAS**	Sum score	0.32 (0.00–0.65)	0.35 (-0.01–0.70)	
	IG	N=74	N=59	0.38 (0.11–0.64)*
	CG	N=74	N=65	0.42 (0.17–0.68)*
**DBS-R**	Overall	0.12 (-0.21–0.44)	0.20 (-0.15–0.56)	
	IG	N=74	N=59	0.41 (0.15–0.68)*
	CG	N=74	N=65	0.26 (0.02–0.51)*
	Ethics	0.14 (-0.19–0.46)	0.10 (-0.26–0.45)	
	IG	N=74	N=59	0.45 (0.18 - 0.71)*
	CG	N=74	N=65	0.37 (0.12 – 0.62)*
	Communication	0.09 (-0.24–0.41)	0.32 (-0.04–0.67)	
	IG	N=74	N=59	0.29 (0.03 – 0.55)*
	CG	N=74	N=65	0.05 (-0.19 – 0.30)
	Lack of control	0.08 (-0.24–0.41)	0.14 (-0.22–0.49)	
	IG	N=74	N=59	0.30 (0.04 – 0.56)*
	CG	N=74	N=65	0.19 (-0.06 – 0.43)
**OHRQoL**	Overall	0.09 (-0.24–0.41)	0.70 (0.33–1.06)*	
	IG	N=73	N=56	0.79 (0.49 - 1.09)*
	CG	N=72	N=64	0.58 (0.31 – 0.85)*
	Global score 1	0.02 (-0.31–0.34)	0.41 (0.05–0.77)*	
	IG	N=74	N=58	0.77 (0.47 – 1.06)*
	CG	N=74	N=65	0.67 (0.40 – 0.94)*
	Global score 2	0.10 (-0.23–0.42)	0.15 (-0.2–0.51)	
	IG	N=74	N=58	0.26 (0.00 – 0.52)
	CG	N=74	N=65	0.23 (-0.01 – 0.48)

APPENDIX

PCPQ	Overall		0.19 (-0.14–0.51)	0.65 (0.28–1.02)*	
		IG	N=73	N=56	0.65 (0.36–0.94)*
		CG	N=72	N=64	0.45 (0.19–0.71)*
	Oral symptoms		0.17 (-0.1–0.49)	0.61 (0.25–0.97)*	
		IG	N=74	N=57	0.81 (0.51–1.11)*
		CG	N=73	N=65	0.70 (0.43–0.97)*
	Functional symptoms		0.31 (-0.02–0.64)	0.52 (0.16–0.89)*	
		IG	N=73	N=56	0.29 (0.02–0.56)*
		CG	N=73	N=64	0.15 (-0.10–0.39)
	Emotional symptoms		0.13 (-0.19–0.46)	0.63 (0.26–0.99)*	
		IG	N=73	N=56	0.62 (0.33–0.90)*
		CG	N=71	N=64	0.30 (0.04–0.55)*
	Social well-being		-0.03 (-0.36–0.29)	0.28 (-0.08–0.64)	
		IG	N=73	N=56	0.42 (0.14–0.69)*
		CG	N=71	N=64	0.24 (-0.02–0.49)
FIS	Overall		-0.17 (-0.50–0.15)	0.57 (0.20–0.93)*	
		IG	N=73	N=56	0.88 (0.57–1.19)*
		CG	N=72	N=64	0.48 (0.22–0.75)*
	Parental and family activities		-0.03 (-0.35–0.30)	0.44 (0.08–0.80)*	
		IG	N=74	N=58	0.71 (0.42–1.00)*
		CG	N=72	N=65	0.45 (0.19–0.71)*
	Parental emotions		-0.34 (-0.66–-0.01)*	0.40 (0.05–0.76)*	
		IG	N=74	N=58	0.81 (0.51–1.11)*
		CG	N=72	N=65	0.36 (0.10–0.61)*
	Family conflict		-0.06 (-0.39–0.27)	0.37 (0.01–0.73)*	
		IG	N=73	N=56	0.38 (0.11–0.65)*
		CG	N=72	N=64	0.15 (-0.11 - 0.40)
	Financial burden		0.08 (-0.24–0.40)	-0.02 (-0.38–0.33)	
		IG	N=74	N=58	0.08 (-0.18–0.34)
		CG	N=74	N=65	0.26 (0.02 – 0.51)*

Note. Between difference is calculated as CG score minus IG score, i.e. positive effect size = CG higher mean score. Within difference is calculated as baseline score minus one-year score, i.e., positive effect estimate = baseline higher mean score.

* p-value < .05

⸸ Cohen's d calculated using the pooled standard deviation

□ Cohen's d calculated using the sample standard deviation of the mean difference

CDAS: Corah Dental Anxiety Scale; CI: confidence interval; CG: control (standard treatment) group; DBS-R: Dental Beliefs Survey; ES: effect size; FIS: Family impact Scale; IG: intervention (oral health coach) group; OHRQoL: Oral Health-related Quality of Life; P-CPQ: Parental-Caregiver Perceptions Questionnaire; SPSQ: Swedish Parenthood Stress Questionnaire.

APPENDIX

Appendix Table 3 Sensitivity analyses: association between treatment intensity (time spent on the phone with the oral health coach) and change in questionnaire scores between baseline and 1-year follow-up, within the intervention group. Each model is controlling for baseline score.

**Measure**	**β-coefficient**	**Standard error**	**p-value**
Overall SPSQ	-0.05	0.04	0.297
CDAS sum score	-0.04	0.28	0.879
Overall DBS-R	-0.11	1.46	0.938
Overall OHRQoL	0.18	1.36	0.897
Overall P-CPQ	0.08	0.98	0.937
Overall FIS	-0.15	0.36	0.679

CDAS: Corah Dental Anxiety Scale; DBS-R: Dental Beliefs Survey; FIS: Family impact Scale; OHRQoL: Oral Health-related Quality of Life; P-CPQ: Parental-Caregiver Perceptions Questionnaire; SPSQ: Swedish Parenthood Stress Questionnaire
